# INCIDENCE OF HIGH GRADE QTCF PROLONGATION AND ITS MANAGEMENT AMONG PATIENTS UNDERGOING TREATMENT FOR DRUG RESISTANT TUBERCULOSIS (DR-TB): CASE SERIES.

**DOI:** 10.21010/ajidv15i2S.5

**Published:** 2021-09-01

**Authors:** Meseret T. Asfaw, David L. Holtzman, Gene F. Kwan, Lawrence T. Oyewusi, Carole D. Mitnick, Kwonjune J Seung

**Affiliations:** 1 Partners in Health, Maseru, Lesotho; 2 Partners in Health, Boston, MA, USA; 3 Harvard Medical School, MA, USA; 4 Boston University School of Medicine, MA, USA

**Keywords:** QT-interval Prolongation, Bedaquiline, Delamanid, Drug Resistant Tuberculosis

## Abstract

**Background:**

The World Health Organization (WHO) has approved the use of two new drugs, namely Bedaquiline (Bdq) and Delamanid (Dlm), for treatment of Drug Resistant Tuberculosis (DR-TB). One of the concerns raised with the use of these drugs was QT-interval prolongation. This condition could be serious and life threatening. Hence, knowing the magnitude and its management is very important. This case series identifies the incidence and discusses the management of clinically significant QT-interval prolongation amongst a cohort of patients who have been on these medicines.

**Materials and Methods:**

Patients with reports of high grade QT-Interval prolongation (i.e. Grade-3 and Grade-4) were identified from the cohort of 265 patients enrolled on bedaquiline and/or delamanid and discussion is made on the pattern, severity and management of each cases identified.

**Results:**

Only 4 (1.5%) out of all 265 patients enrolled on Bedaquiline and/or Delamanid have developed high grade QT-Interval prolongation. And all are managed without permanent discontinuation of both drugs.

**Conclusion:**

The Incidence of clinically significant QTcF-interval prolongation among DR-TB patients taking bedaquiline and /or delamanid in Lesotho is low. And almost all cases can be managed with more frequent Electrocardiogram (ECG) monitoring and management of other possible causes of QT-interval prolongation without the need to stop one or both drugs permanently.

## Introduction

There were an estimated 500,000 new cases of rifampicin-resistant TB globally in 2018. Three hundred ninety thousand had multidrug-resistant TB (MDR-TB) and 6.2 % of this were extensive drug-resistant (XDR) TB (World Health Organization, 2019). Older DR-TB medicines and regimens are associated with many side effects with low treatment success rates (Jennifer Furin et al., 2015). The approval and introduction of bedaquiline (Bdq) in 2012, delamanid (Dlm) in 2013 and repurposing of linezolid (Lzd) and clofazimine (Cfz) has improved DR-TB treatment outcomes (Expand New Drugs for TB, 2015).

QT interval prolongation is one of the adverse effects of concern with the use of these drugs, particularly for Bdq and Cfz and to a lesser degree Dlm. (Expand New Drugs for TB, 2015). In addition, other QT-interval prolonging medicines are also frequently used along with these drugs to treat co-morbidities and manage certain side effects of DR-TB (WHO, 2014). Previous publications have reported rates of 15% for QT-interval increase of ≥30 msec above baseline, 26% for >1 episode of QT-interval >450 msec, and 5% occurrence of QT-interval ≥500 msec with use of Bdq and Dlm for varying durations (endTB Clinical and Programmatic Guide, 2016: Mbuagbaw et al., 2017).

Here we are reporting a case series of four patients with significant QT prolongation among a cohort of DR TB patients treated with bedaquiline and/or delamanid in Lesotho.

## Materials and Methods

This case series was extracted from Lesotho site data of The Expanding New Drugs for TB (endTB) observational study (Khan et al 2019), a multi-country, prospective cohort study of confirmed RR/MDRTB patients that received at least 6 months of Bdq and/or Dlm topped to the standardized MDR TB regimen of the country and the study was funded by Unitaid.

Participants who fulfilled the inclusion criteria were enrolled to the cohort after providing their informed consent (Expand New Drugs for TB, 2015) and were monitored with 12-lead electrocardiograms (ECGs) performed at baseline, 2 weeks on treatment and every month thereafter for the duration of treatment with Bdq or Dlm. The duration of treatment with Bdq or Dlm was determined by the study physicians according to a patient’s response to treatment, but typically lasted for 6 months. The ethical approval was received on 7^th^ of September, 2015 and the study was conducted in accordance to the ethical standards established by the Lesotho National Health and Research Ethics Committee and the Helsinki Declaration on the ethical principles of human subjects research.

The QT-interval, corrected with Frederica’s formula (QTcF), was calculated by study physicians and graded as follows (Expand New Drugs for TB, 2015): normal <450 msec, grade 1 450-480 msec, grade 2 481-500 msec, grade 3≥501 msec without cardiac symptoms, and grade 4 ≥501 msec or >60 msec increase from baseline with cardiac symptoms. Participants with grade 3 and 4 QTcF prolongation were identified and sent for review by the study cardiologist. Consequently, four (1.5%) participants out of 265 were confirmed to have grade 3 or 4 QTcF prolongation, which are summarized in Table 1 below.

**Table 1:** Summary table of patients with QT prolongation.

*Cases*	*Baseline QTcF (msec)*	*Initial regimen*	*Longest QTcF (msec)*	*Time of prolongation (days)*	*Likely cause of QTcF prolongation*	*Managements made*	*Final regimen*	*Final treatment outcome*
***1***	416	Dlm-Lfx-Cfz-Cs-Pto	523	32	Clofazimine	Cfz stopped	Z-Lfx-Pto-Cs	Cured
***2***	345	Bdq-Dlm-Lzd-Cfz-PAS	488	136	Baseline cardiac disease	Cardiac failure managed	Bdq-Dlm-Lzd-Cfz	Cured
***3***	421	Dlm-Lfx-Lzd-Cfz-Km	518	23	Clofazimine	Cfz stopped	Lfx-Lzd-Cs-Pto	Cured
***4***	429	Bdq-Lfx-Cs-Pto-PAS-Z	536	29	Electrolyte disorder (Hypokalemia)	Potassium given	Lfx-Lzd-Cs-Dlm-Pto	Cured

**Bdq-** Bedaquiline, **Cfz-** Clofazimine **Cs**-Cycloserine, **Dlm-** Delamanid, , **Km-** Kanamycin, **Lfx-**Levofloxacin, **Lzd-**Linezolid **PAS**-Para Amino salicylic Acid and **Pto**- Prothionamide

## Cases

The first case is a 57-year-old known Human Immunodeficiency Virus (HIV)-positive and chronic obstructive pulmonary disease (COPD) patient who was relatively stable and on treatment for both conditions. He was diagnosed with MDR-TB in January 2017 after two prior successful treatment courses for drug-susceptible TB in 2014 and 2018 respectively. He was started on a MDR-TB regimen of levofloxacin-delamanid-prothionamide-cycloserine and clofazimine after inpatient stabilization of initial respiratory distress. His baseline QTcF-interval was 416 msec but increased to 523 msec after one month of MDR-TB treatment. Patient was not having any cardiac symptoms, did not have any evidence of electrolyte disorders, organ injury or endocrine abnormalities on laboratory tests and was not taking any concomitant medicines known to prolong the QT-Interval as well (Rohan J et al., 2002). Hence, he was observed for spontaneous resolution with frequent ECG monitoring. Despite this the QTcF persisted above 500 msec and finally clofazimine was stopped after 10 days of observation. Follow up QTcF recorded on wards kept on falling and normalized after a week of clofazimine discontinuation. Afterwards, the patient never had prolonged QTcF for the rest duration of treatment until discharged with an outcome of cure at 18 months.

The second patient is a 37-year-old HIV-positive male with XDR-TB. He was initiated on an individualized regimen of pyrazinamide (Z)-bedaquiline-delamanid-linezolid-clofazimine and para-amino salicylic acid (PAS) in January 2017. The baseline ECG was normal with QTcF-interval of 345 msec. He was admitted to hospital after five months of treatment with acute congestive heart failure (CHF). An ECG done on admission showed sinus rhythm with QTcF-interval of 488 msec. Bdq, Dlm, Z and PAS were all withheld because of the CHF, QTcF-interval prolongation, and mild elevation in liver enzymes on lab investigations. The patient responded well to CHF management and the QTcF-interval normalized within one week. The MDR-TB regimen was modified to ethambutol-linezolid-clofazimine-delamanid for the next three months after which bedaquiline was reintroduced. The patient took a Bdq and Dlm containing regimen for the next 11 consecutive months at which point he had completed 19 months of total duration of treatment and was discharged from treatment with an outcome of cure. As a result, the patient’s QT-interval prolongation was likely to have resulted from the CHF, PAS or other factors.

The third case is a 25-year-old HIV-negative male patient who was diagnosed with RR-TB and initiated on levofloxacin-kanamycin-delamanid-linezolid-clofazimine. The baseline QTcF-interval was 421 msec but prolonged to 518 msec after 23 days of treatment. No laboratory abnormality was identified and he was not taking any concomitant medications thought to prolong the QT-Interval as well, so, clofazimine was stopped and ECG monitoring continued. The ECG repeated after a week recorded a sinus rhythm with QTcF 463 msec. Monthly ECG recordings were done for the rest of patient’s treatment course and no ECG above 500 msec was recorded then after.

The fourth case is a 22-year-old HIV-negative female patient diagnosed with MDR-TB and initiated on levofloxacin-bedaquiline-prothionamide-cycloserine-para-aminosalicylic acid and pyrazinamide. After taking the above regimen for a month, the patient presented to the Hospital with 5 days’ complaints of vomiting and admitted with diagnosis of Dehydration secondary to fluid loss from vomiting due to gastrointestinal upset and to rule out liver injury. All TB medications were stopped, blood sample drawn for chemistry test and patient was rehydrated. ECG done on the next day showed a sinus rhythm with QTcF of 536 msec. Blood tests revealed only mild elevation of liver enzyme (ALT=68 u/L and AST=Normal) with mild hypokalemia (3.46 meq/l). The ECG abnormality resolved few days later after rehydrating patient, and the same regimen re-started, with no repeat record of prolonged QT over two weeks’ period. However, the liver enzymes kept on rising reaching (ALT= 207 u/L and AST=171 u/L). As a result, possible hepatotoxic drugs (i.e. Pyrazinamide, Bedaquiline, and Para-aminosalicylic acid) were permanently stopped. Delamanid was added making a regimen containing Levofloxacin-Prothionamide-cycloserine-Delamanid-Linezolid. The patient took this regimen without any problem for 20 months and finished her treatment with an outcome of cure.

## Discussion

Out of 265 cases of DR-TB enrolled to either Bdq, Dlm or both over a course of 3 years, only four patients had grade-4 or clinically significant QTcF-interval prolongation (i.e. above 500 msec or 60 msec above baseline with cardiac symptoms). In addition to taking either Bdq and/or Dlm, each of the cases was receiving at least one other DR-TB drug presumed to prolong the QT-interval (clofazimine or levofloxacin) (Guglielmetti et al., 2018) or both in their DR-TB regimen. The mean number of days to detection of QTcF prolongation was 28 days. Similar to findings from other settings (Pontali et al., 2017: Udwadia et al., 2014), none of the patients had serious life-threatening cardiac symptoms. The longest QTcF-interval recorded was 536 msec and it happened after one month of treatment .Two cases were managed with permanent discontinuation of clofazimine but no patient permanently stopped Bdq or Dlm as a result of QTcF-interval prolongation.

## Conclusion

The Incidence of clinically significant QTcF-interval prolongation among DR-TB patients taking new and repurposed drugs is low in Lesotho. Clofazimine could be a more significant contributor to QTcF-interval prolongation. Regular ECG monitoring and prompt and aggressive management of comorbidities and side effects remains important for picking and correcting any QTcF prolongation. In a nutshell, the risk of QTcF prolongation due to new DR-TB drugs is very low in Lesotho though cessation of routine ECG monitoring is not warranted.

**Conflict of Interest/Competing Interest**: The authors declared that there is no conflict of interest associated with this study.

**Abbreviations:**
ALT-: Alanine Transaminase
AST-: Aspartate Transaminase
Bdq-: Bedaquiline
Cfz-: Clofazimine
CHF-: Congestive Heart Failure
COPD-: Chronic Obstructive Pulmonary Diseases
Cs-: Cycloserine
Dlm-: Delamanid
DR-TB-: Drug Resistant Tuberculosis
ECG-: Electrocardiogram
HIV-: Human Immunodeficiency Virus
Lfx-: Levofloxacin
Lzd-: Linezolid
MDR-TB-: Multi-Drug resistant TB
PAS-: Para-amino Salicylic Acid
Pto-: Prothionamide
RR-TB-: Rifampicin Resistant TB
TB-: Tuberculosis
XDR-TB-: Extensively-Drug Resistant TB.

## References

[1] Emanuele Pontali Giovanni Simon Lia D'Ambrosio Rosella Centis and Giovanni B. Migliori Tiberi (2017). Cardiac safety of bedaquiline:a systematic and critical analysis of the evidence. European Respiratory Journal.

[2] endTB (Expand New Drugs for TB) Observational Study (2015). Protocol for Treatment of MDR-TB with regimens containing bedaquiline or delamanid. Version 2.0.

[3] endTB Clinical and Programmatic Guide for Patient management with new TB Drugs. 2016. Virsion3.3:9.

[4] Guglielmetti L, Barkane L, Le Dû D, Marigot-Outtandy D, Robert J, Veziris N (2018). Safety and efficacy of exposure to bedaquiline-delamanid in multi-drug resistant tuberculosis:a case series from France and Latvia. European Respiratory Journal.

[5] Jennifer Furin, Grania Brigden, Erica Lessem, Michael Rich, Laura Vaughan, Sharonann Lynch (2015). Global Progress and Challenges in Implementing New Medications for Treating Multidrug-Resistant Tuberculosis. Emerging Infectious Disease.

[6] Lawrence Mbuagbaw (2017). Review of available evidence on the use of bedaquiline for the treatment of multidrug-resistant tuberculosis March.

[7] Rohan J, Pramesh K (2002). Drugs and the QTc interval. Australian Prescriber.

[8] Uzma Khan (2019). The endTB observational study protocol:treatment of MDR-TB with bedaquiline or delamanid containing regimens.

[9] World Health Organization (2019). Global Tuberculosis Report.

[10] World Health Organization (2014). Companion Hand book to the WHO guidelines for programmatic management of drug-resistant tuberculosis.

[11] Udwadia Z. F, Amale R. A, J. B (2014). Mullerpattan.Initial experience of bedaquiline use. in a series of drug-resistant tuberculosis patients from India. INT J TUBERC LUNG DIS.

